# Preface from the Guest Editor of Special Issue “Quantum-Chemical Modeling and Design of Chelate and Macrocyclic Metal Complexes”

**DOI:** 10.3390/ijms21072339

**Published:** 2020-03-28

**Authors:** Oleg V. Mikhailov

**Affiliations:** Analytical Chemistry, Certification and Quality Management, Kazan National Research Technological University, K. Marx Street 68, 420015 Kazan, Russia; olegmkhlv@gmail.com

As is known, in the last fifty years of coordination of inorganic and organo-elemental chemistry, chemical compounds formed by chelating or macrocyclic organic and organo-elemental ligands (in particular, crown ethers, cryptands, calixarenes, cucurbituryls, porphyrins, and their various derivatives) and ions of various *p*-, *d*- and *f*-elements, have become of special interest. These compounds, collectively called “macrocyclic metal complexes” or “macrocyclic metal chelates”, have a number of specific (and sometimes, unique) properties that are not inherent in those organic and organo-elemental compounds that make up their composition. As a result of this, they have already found and continue to find a lot of applications. The list of industries where they are now in demand, includes metallurgy and medicine, industrial biotechnology and catalysis, microelectronics and agriculture, and many other branches of human activity. In recent years, they have also found use in molecular nanotechnology. In this context, macrocyclic metal complexes are those objects to which close attention is now being given not only be chemists, but also researchers from other branches of science and practice, both from different countries of the world, and from a very different profile.

Macrocyclic metal complexes are distinguished by one important feature among other metal complexes, namely the presence of three or more (articulated) metal chelate rings connected to each other. Each of these rings contain at least two atoms that are part of two adjacent metal chelate cycles, and for this reason, they occupy a special position in modern chemistry and especially in coordination chemistry. At the present time, macrocyclic metal complexes are usually divided into two categories, namely, metal complexes with the so-called open contour and metal complexes with the so-called closed contour. The first of these categories includes such metal complexes in which the complexing metal atom is located on the “rim” of the so-called macrocycle (a closed group of atoms, which, according to the definition adopted by chemists today, contains at least nine atoms, including at least two heteroatoms) and is necessarily one of those atoms that form this macrocycle. Macrocyclic metal complexes of the given type contain so-called compartmental ligands, which, as a rule, are acyclic polydentate organic compounds characterized by a specific spatial arrangement of donor atoms responsible for the formation of “pre-organized chambers”, for coordination of one or more ions of *p*-, *d*-, or *f*-elements. It should be noted in this context, that the formation of these “chambers” requires a certain (and usually very significant) distortion of the molecular structure of the corresponding compartmental ligand forming the metal complex. Additionally, in almost all currently available studies on macrocyclic metal complexes with open contour, these compartmental ligands were tetradentate, and as a rule their compounds contained three articulated metal chelate rings and belonged to the so-called macro-tricyclic metal complexes. The second category includes macrocyclic metal complexes in which the complexing metal atom itself does not participate in the formation of the macrocycle, but is inside it in a type of “chelate cell” formed by a macrocyclic ligand (i.e., one that initially contains at least one macrocycle). Unlike macrocyclic metal complexes of the first category, metal complexes of this second category contain four metal chelate cycles (or an even greater number of them) and, therefore, belong to the so-called macro-tetracyclic metal complexes. It should be noted in this context, that during the formation of such compounds, the molecular structure of the macrocyclic ligand forming the metal complex either does not change at all or only undergoes minor changes. This special issue includes articles on macrocyclic metal complexes of both of the above categories. Taking into account the given important circumstance, the present special issue includes articles devoted to macrocyclic metal complexes of both of the above categories.

Macrocyclic metal complexes, irrespective of whether they belong to the first or the second of the above categories, can also be systematized by their assortment of articulated metal chelate rings and, first of all, by the number of atoms in them, which can be indicated in brackets as digital indices; the number in these indicators corresponds to the total number of chelate rings. In the framework of such a system, a macro-tricyclic metal complex containing two five-membered and one seven-membered metal chelate ring, should be denoted by the indices (575), (557), or (755); a macro-tetracyclic metal complex with two four-membered and two six-membered cycles, should have the indices (4646), (6464), (4664), (6446), (4466), or (6644), depending on the sequence of the arrangement of the corresponding metal chelate rings, with different number of atoms in the molecule of the macrocyclic metal complex and in the numbering order of these rings. In turn, within each of these categories of macrocyclic metal complexes, two more subcategories can be distinguished, namely the so-called symmetric and asymmetric complexes; the first metal complex has a plane of symmetry perpendicular to that plane in which the structural formula of the given complex is depicted, while the second metal complex does not have such a plane. Wherein, in asymmetric complexes, the sets of atoms in metal chelate rings with the same number of members can be the same or different. Other variants of the systematics of macrocyclic metal complexes are also possible, by changing the coordination number of the complexing metal ion, by changing the nature of the donor atoms of the compartmental or macrocyclic ligand, etc. 

The first of these unique compounds, copper(II) phthalocyanine, was accidentally obtained by Japanese chemists almost 100 years ago, in 1927 (although none of them realized it at the time that they had obtained namely a macrocyclic metal complex). Whereas the second compound (as it is not rare) was discovered only after forty years later, in 1961. In this connection, it is noteworthy (and even symptomatic) that in both of these cases macrocyclic metal complexes were obtained as a result of very specific chemical reactions, namely the so-called “self-assembly”, when at least one of the ligands contained in such a complex, was formed from simpler “building blocks”, and this self-assembly occurred only in the presence of certain metal ions. In modern coordination chemistry, more specific terms are used to refer to such “self-assembly”, namely “template synthesis”, “template process”, or “template reaction”. In general, template synthesis is now understood as a variety of complex formation reactions, where a metal ion or other reaction center with a certain stereochemistry and electronic structure acts as a pattern, template, form, or matrix, for the formation, under the conditions of any reaction of the only possible or predominant product from the corresponding starting substance. Additionally, synthesis of this product under other conditions is either extremely difficult or cannot be realized at all. Such a synthesis includes two aspects—the formation of the ligand system due to the “organizing” and “directing” roles of the metal complexing ion, on the one hand; and the reaction of organic compounds associated with this ion (the reaction of coordinated ligands), on the other hand. In this regard, it is worth noting that the processes of “self-assembly” quite often turn out to be key in the synthesis of such macrocyclic compounds that do not contain metal atoms; in this case, the macrocyclic metal complexes formed initially are subjected to demetallation by means of any additional chemical reactions. Template processes and their associated reactions have attracted the special attention of specialists in the field of chemistry of macrocyclic compounds, primarily because in the vast majority of cases they lead either to the appearance of additional metal chelate rings or to the crosslinking of cyclic groups previously existing in the complex, into a unified closed contour. However, almost any of these processes usually proceed under rather “hard” conditions and require applying an elevated temperature to the reaction system during a very long (several hours or even more) time. This is partly due to the fact that, when constructing macrocyclic compounds from individual structural fragments (i.e., ligand synthons), a very significant decrease in entropy is associated with a decrease in the number of rotational and vibrational degrees of freedom in the system (as a result, the likelihood of formation of reaction products containing closed contours of atoms is also reduced). This process could be greatly facilitated if carried out in the so-called organizing systems that promote structural reorganization of chemical compounds arising during the reaction, and, in particular, coordination compounds. Nevertheless, by the end of the 20th century, the number of publications devoted to the synthesis and study of the physicochemical properties of macrocyclic metal complexes, exceeded 1000; at the same time, they included not only original articles and reviews but also included special monographs. In this connection, it should be noted especially that the vast majority of these compounds (both open and closed contour) include *d*-element atoms and polydentate organic ligands with donor atoms of nitrogen, oxygen, and sulfur. At the present time, template synthesis is also one of the most important synthetic techniques in supramolecular chemistry, where macrocyclic compounds also occupy a prominent place.

For predicting both the physicochemical properties of various macrocyclic metal complexes and controlling the processes of their synthesis (“self-assembly”), information on their molecular structures and its key parameters (bond lengths, valence, and torsion (dihedral) angles) is very important. However, in a number of cases, the determination of the indicated parameters for such chemical compounds presents very significant difficulties, since macrocyclic metal complexes cannot always be isolated from the reaction system in the form of single crystals suitable for carrying out X-ray diffraction analysis. Such a situation, in particular, arises in those cases when the “self-assembly” of these compounds is carried out in any polymer matrix, for example, in a gelatin mass. Owing to this, prediction of their physicochemical parameters determining these very properties, is of great importance. Such a problem is currently being successfully solved thanks to the presence of both modern quantum-chemical methods of calculation (like the Density Functional Theory (DFT) method), as well as computer technologies and the corresponding experimental equipment. Additionally, a number of such parameters that are important for understanding the physicochemical nature of these compounds but which are difficult or impossible to obtain at this level of development of experimental technique (in particular, the distribution of electron density and the charges on the individual atoms included in the metal complex that are connected with it, the values of the relative energy of the ground and excited states, and the assessment of the stability of molecular structures) can simultaneously be determined. At the same time, there are relatively few theoretical works devoted to quantum-chemical calculations of the above metal complexes through the DFT method, and, even more, that use more advanced quantum-chemical methods. In any case, there are much fewer of such studies than there are works devoted only to the synthesis of these compounds. To an extent, the reason for this is the complexity of the molecular structures of macrocyclic metal complexes, as a result of which, the quantum-chemical calculation of these compounds using even the DFT method with the simplest basic sets (not to mention more advanced theoretical methods) is in most cases time-consuming, and therefore, is very difficult for practical implementation. Nevertheless, there is no doubt that, with improvement of computer technique and technologies, this method will become more accessible for use in scientific work, including macrocyclic metal-containing compounds. The given special issue of the *International Journal of Molecular Sciences* is designed to, at least to some extent, fill this still existing gap in the chemistry of macrocyclic compounds.

